# On the fate of seasonally plastic traits in a rainforest butterfly under relaxed selection

**DOI:** 10.1002/ece3.1114

**Published:** 2014-06-04

**Authors:** Vicencio Oostra, Paul M Brakefield, Yvonne Hiltemann, Bas J Zwaan, Oskar Brattström

**Affiliations:** 1 Institute of Biology, Leiden UniversityPO Box 9505, 2300 RA, Leiden, the Netherlands; 2 Laboratory of Genetics, Wageningen University and Research CentreP.O. Box 309, 6700 AH, Wageningen, the Netherlands; 3 Department of Zoology, University of CambridgeDowning Street, Cambridge, CB2 3EJ, UK

**Keywords:** *Bicyclus anynana*, *Bicyclus martius*, *Bicyclus sanaos*, constraints, life-history evolution, phenotypic plasticity, reproductive investment, seasonality

## Abstract

Many organisms display phenotypic plasticity as adaptation to seasonal environmental fluctuations. Often, such seasonal responses entails plasticity of a whole suite of morphological and life-history traits that together contribute to the adaptive phenotypes in the alternative environments. While phenotypic plasticity in general is a well-studied phenomenon, little is known about the evolutionary fate of plastic responses if natural selection on plasticity is relaxed. Here, we study whether the presumed ancestral seasonal plasticity of the rainforest butterfly *Bicyclus sanaos* (Fabricius, 1793) is still retained despite the fact that this species inhabits an environmentally stable habitat. Being exposed to an atypical range of temperatures in the laboratory revealed hidden reaction norms for several traits, including wing pattern. In contrast, reproductive body allocation has lost the plastic response. In the savannah butterfly, *B. anynana* (Butler, 1879), these traits show strong developmental plasticity as an adaptation to the contrasting environments of its seasonal habitat and they are coordinated via a common developmental hormonal system. Our results for *B*. *sanaos* indicate that such integration of plastic traits – as a result of past selection on expressing a coordinated environmental response – can be broken when the optimal reaction norms for those traits diverge in a new environment.

## Introduction

Phenotypic plasticity is the ability of a particular genotype to express different phenotypes in response to environmental variation (Schlichting and Pigliucci 1998; West-Eberhard 2003). Although not necessarily adaptive, many instances of adaptive phenotypic plasticity have been documented. In these cases, organisms expressing distinct phenotypes in alternative environments have their highest relative fitness in the environment in which they typically occur (see Stearns 1989; Beldade et al. 2011; Simpson et al. 2011). In seasonal habitats, phenotypic plasticity may evolve as a result of contrasting but predictable seasonal selection pressures, resulting in different morphologies and/or life-history strategies being expressed in each season (Shapiro 1976; Brakefield and Zwaan 2011).

It has rarely been studied what the evolutionary fate of plasticity would be in an ancestrally plastic species that no longer inhabits such a seasonal environment so that the traits are no longer exposed to environmental variation previously associated with their plasticity (Lahti et al. 2009; Snell-Rood et al. 2010; but see Aalberg Haugen et al. 2012). This could occur when a species adapted to a seasonally fluctuating habitat establishes itself in a new, aseasonal habitat, or when the environmental conditions within a habitat change such that it becomes less seasonal. In such circumstances, part of the previous phenotypic range of this trait is no longer expressed on a regular or predictable basis and therefore not exposed to natural selection. Such relaxed selection also applies to plasticity for the trait, that is, the ability to express other mean trait values in response to environmental variation.

The question whether relaxed selection will result in loss or retention of seasonal plasticity for a particular trait will depend on the interplay between benefits, costs (both direct and indirect via pleiotropy), and neutral processes such as mutation accumulation and genetic drift (Lahti et al. 2009). Retaining plasticity in a less seasonal environment might be favored through direct benefits if the environment still shows limited variability, but of a less regular nature. Indirect benefits might be due to pleiotropic effects of seasonal plasticity on correlated traits that are still under strong selection. This might be the case if genetic or developmental mechanisms regulating seasonal plasticity are also involved in other, nonseasonal environmental responses such as responses to diurnal temperature fluctuations. If there is no such pleiotropy, mutations in genes responsible for seasonal plasticity can accumulate, potentially, but not necessarily, leading to reduction or loss of the seasonal response (Lahti et al. 2009; Aalberg Haugen et al. 2012). Finally, plasticity, when under relaxed selection, might be reduced or lost due to two proposed types of costs of plasticity. First, there can be direct ‘production’ costs of expressing a particular trait, as in the case of energetic costs of inducible anti predatory defenses. Second, theoretical considerations suggest the existence of inherent costs associated with the capacity to produce different phenotypes under different conditions. The costs of this capacity presumably lie in the maintenance of sensory and regulatory systems needed to sense environmental conditions and translate them into phenotypic alterations. However, empirical studies suggest that such plasticity costs are generally weak, making it unclear whether they would play a large role in evolutionary loss of plasticity under relaxed selection (Callahan et al. 2008; van Buskirk and Steiner 2009; Auld et al. 2010). In general, empirical studies on the evolutionary consequences of relaxed selection on plasticity are relatively rare (Lahti et al. 2009; Snell-Rood et al. 2010; Schwander and Leimar 2011), especially in the context of plasticity as adaptation to seasonally fluctuating environments (but see Aalberg Haugen et al. 2012). Here, we study whether a phylogenetically widespread and likely ancestral capability to respond to seasonal environmental variation is still retained in a species that inhabits a less seasonal habitat, where natural selection on plastic responses is assumed to be relaxed.

Seasonal plasticity of wing pattern occurs frequently in the Nymphalidae butterfly family, for example in *Melanitis leda* (Brakefield and Larsen 1984), *Junonia coenia* (Rountree and Nijhout 1995), and *Araschnia levana* (Windig and Lammar 1999). Seasonal plasticity, often with discrete phenotypes in the wet and dry season, is well documented in the Subtribe Mycalesina (Satyrinae), which inhabit a multitude of habitats in the old world tropics (Brakefield and Reitsma 1991; Braby 1994; Brakefield and Frankino 2009). Particularly well studied among Mycalesina is the genus *Bicyclus* (Kirby, 1871), where seasonal plasticity in wing pattern is very common. The genus comprises of *ca*. 90 species distributed throughout sub-Saharan Africa, inhabiting savannah-woodland as well as rainforest habitats (Condamin 1973; Brakefield and Frankino 2009). In the dry season in seasonal habitats, adult *Bicyclus* butterflies typically express a cryptic wing pattern allowing them to rest undetected among the dried out vegetation. In the wet season, vegetation is green and abundant and the adults of the same species now instead express prominent concentric eyespots along the distal margin of their wings. These dry and wet season morphs are expressed by separate cohorts of butterflies, and the adult wing pattern is fixed at emergence. The eyespots are probably involved in deflecting vertebrate predator attacks away from the vulnerable body toward the margin of the wing (Lyytinen et al. 2003, 2004; Brakefield and Frankino 2009). Given that seasonal polyphenism is widespread throughout the whole Subfamily (Satyrinae), and that most species of *Bicyclus* (even those inhabiting evergreen forest) show signs of plasticity, this is likely the ancestral state for the group (Brakefield and Frankino 2009). The most studied species is *Bicyclus anynana* (Butler, 1879), a savannah-woodland butterfly distributed throughout East Africa (Brakefield et al. 2009). In addition to wing pattern, *B. anynana* adults of the wet and dry seasons differ markedly in their life-history strategies. In the field, adults spend the harsh dry season being relatively inactive and delay reproduction until the beginning of the wet season, when larval food plants reappear. The relatively short-lived adults of the wet season morph are more active and reproduce rapidly. Normally, two to three such generations occur in the wet season. In the laboratory, wet season females allocate relatively more mass to the abdomen (Oostra et al. 2011) and lay more (albeit smaller) eggs (Brakefield and Zwaan 2011).

At a proximate level, the major cue for the induction of adult dry or wet season phenotypes is the temperature experienced during the late larval and early pupal stages. At high temperatures, corresponding to wet season conditions in the field, *B. anynana* larvae develop into wet season adults, whereas low temperatures, indicative of an approaching dry season in the field, induce development of the dry season morph (see Oostra et al. 2011). Both adult wing pattern and life history are determined by developmental temperature, although life-history traits retain the ability to acclimatize over the course of several weeks when environmental conditions change during adult life (e.g., Fischer et al. 2003). Recently, we showed that Ecdysteroid hormones during the pupal stage play a functional role in regulating developmental plasticity of adult reproductive strategy, a role for this hormone that had already been established for wing pattern plasticity (Oostra et al. 2011b). This indicates that, at least in *B. anynana*, developmental plasticity of wing pattern and of life history share developmental-physiological mechanisms. Such developmental integration of both forms of plasticity may have been driven by the correlated fluctuations in selection pressures on both wing pattern and life history between the seasons.

To examine the evolutionary effects of relaxed selection on developmental plasticity of wing pattern and life history, we used *Bicyclus sanaos* (Hewitson, 1866) (see method for discussion about species name issues), a rainforest species that is only rarely exposed to dry season like conditions in the field. *B. sanaos*' closest relatives are all forest species, and show some level of wing pattern plasticity. However, only two of the related forest species can be classified as possibly seasonally polyphenic (*B. istaris* and *B. sophrosyne*). At the basal branching of the same clade of *Bicyclus* is one species group whose members are among the most seasonal savannah species of the whole genus (*B. cottrelli* and *B. safitza*) (Brakefield and Frankino 2009). The available phylogenetic data suggest that the ancestors of *B. sanaos* left the savannah habitat about 10 million years ago (Monteiro and Pierce 2001). This long-term lack of seasonal exposure to harsh dry season conditions likely reflects a situation of relaxed selection on plasticity.

The aim of this study was to investigate to what extent *B. sanaos* has retained the ability to express alternative phenotypes when exposed in the laboratory to a range of ‘seasonal’ temperatures not normally encountered in the field. Using a recently established laboratory population, we measured the thermal responses for a suite of traits that in *B. anynana* are plastic and involved in the seasonal adaptation. This was done on parallel cohorts of developing larvae, across a temperature range that in other *Bicyclus* species induce plasticity (Roskam and Brakefield 1996). The traits investigated included life-history traits, physiological traits, and wing patterns. We then compared thermal responses to those observed in a previous reaction norm experiment in *B. anynana* (Oostra et al. 2011). Our experiment allowed us to determine not only the thermal plasticity of each individual trait but also the extent to which these traits show an integrated response to the environment comparable to that in *B. anynana*. Performing a comparative study of plasticity responses using only two species is a limitation, but to our knowledge this is the first time a reaction norm study with detailed measurements of several traits has been conducted using nonmodel species of butterflies.

## Materials and Methods

### *Bicyclus sanaos* habitat and laboratory population

*Bicyclus martius sanaos*, in several previous publications called *B. sanaos* (Larsen 2003), is fairly common in African rainforests, but never found in open savannah habitats. Ongoing revisional work on the taxonomy of the whole genus *Bicyclus* shows that this subspecies of *Bicyclus martius* is indeed a valid species of its own (O. Brattström, H-L. Wang, K. Aduse-Poku, C. Löfstedt and P. M. Brakefield, unpubl. data). In this article, we are therefore using the name *Bicyclus sanaos* when referring to what is still currently officially named *Bicyclus martius sanaos*. The laboratory stock was established in the laboratory in Leiden from 60 gravid females collected in Ologbo Forest (N 6.02, E 5.55, 20 m.a.s.l.) in southern Nigeria. On this location, temperature varies very little throughout the year (25–28°C mean monthly temperature), but precipitation shows marked seasonality (30–450 mm per month; as measured at a weather station ca. 60 km away (N 5.52, E5.73; National Climatic Data Center). Despite the variation in precipitation, the soil remains wet during the whole dry season, the vegetation in the forest interior remains green and the humidity stays high throughout the year. Thus, larval food plants (i.e., grasses) are likely to be continuously available. Furthermore, *B. sanaos* adults of all ages can be observed at any given time, from recently eclosed individuals with no visible wing wear through to old individuals with extensive wing damage (O. Brattström, pers. obs.). This suggests that females breed throughout the year and show no seasonal suppression of reproductive activity. Occasionally, individuals with small ventral eyespots, resembling a typical *Bicyclus* dry season morph, are found in the field. However, the majority of individuals have large, wet season-like eyespots all year round, including during the dry season (O. Brattström, pers. obs.; Roskam and Brakefield 1999). This contrast strongly with the natural savannah habitat of *B. anynana* that shows a high annual variation in mean monthly temperature (17–25°C) and a marked seasonality in rainfall (0–290 mm per month). More importantly, most years will see at least three consecutive months with no rainfall at all meaning all available food plants for larvae will dry out for a prolonged time (National Climatic Data Center).

Overall, the butterfly rearing setup for the stock of *B. sanaos* in the laboratory was comparable to that used in *B. anynana* (see Brakefield et al. 2009), with a slightly higher temperature (28°C) and relative humidity (RH; 85%). The only major difference compared to *B. anynana* was the larval food plants. Adult females oviposited, and larvae grew, on young pot-grown *Oplismenus sp*., and *Triticum sp*. (wheat) plants (both Poaceae). Each generation, about 400 larvae were reared, of which between 40 to 60% normally survived through to adulthood. Females generally started ovipositing between 2 to 3 weeks after eclosion, and continued to do so at a relatively constant rate for several weeks. Adults regularly survived longer than 100 days after eclosion.

### Experimental design and measurement of phenotypic responses

We assessed developmental plasticity in *B. sanaos* by rearing separate cohorts of larvae at three different temperatures and measuring phenotypic responses for life history and wing pattern traits. We collected eggs from the stock population and allowed the larvae to hatch on *Triticum* (wheat) plants. The freshly hatched larvae were collected on a daily basis and transferred in batches of 20 larvae onto separate one-week-old *Triticum* plants kept in individual net sleeves, which were each placed in one of three environmental climate chambers set at 19, 23, or 27°C (and 85% RH with a 12:12 L:D photoperiod). We placed a total of 10 such sleeves in each climate chamber, rearing 200 larvae per temperature. Plants were monitored daily and watered or replaced as necessary. Pre-pupae were collected daily and placed in Petri dishes to pupate. One-day-old pupae were weighed to the nearest 0.01 mg using a Sartorius Research RC 210D scale, and then placed in individual pots until eclosion. Larval and pupal development times were recorded in days. Subsequently, adult resting metabolic rate (RMR) was measured as the individual rate of CO_2_ respiration (mL·h^−1^) over a period of 20 min, at 27°C during the dark phase of the diurnal cycle (following Pijpe et al. 2007). Butterflies were then frozen at −20°C until further processing. Abdomens and thoraces (removing head, wings, antennae, and legs) were then dried to constant mass for 48 h at 60°C before being weighed separately. One ventral hindwing of each adult was imaged using a Leica M125 stereo microscope coupled to a Leica DFC495 digital camera. In *B. sanaos*, the basic wing pattern elements on the ventral wing surfaces are similar to those in *B. anynana*: a series of concentric eyespots along the distal margins of the fore and hind wing (Roskam and Brakefield 1996). We used ImageJ software v1.46 r (Abramoff et al. 2004) to measure three characteristics of the ventral wing pattern on the digital image of each hindwing: radius of the second eyespot (starting from anterior), radius of the fifth eyespot, and distance between the center of the second and the fifth eyespot (as a measurement of wing size). Eyespot radii were measured from the center of each eyespot's white focus to the most proximal point on the outer boundary of the golden ring.

### Comparison with *Bicyclus anynana*

We compared the thermal responses in *B. sanaos* to those of *B. anynana* using data on developmental plasticity in *B. anynana* from a previous reaction norm experiment (Oostra et al. 2011). In this experiment, *B. anynana* larvae were also reared at 19, 23 and 27°C and phenotypic responses in the adults were measured exactly as for *B. sanaos*. The only differences between the experiments were the food plants (*B. anynana* larvae were fed *Zea mays* plants rather than *Triticum*; see Brakefield et al. 2009), and the use of a slightly lower humidity (70%) for *B. anynana*. We know that the choice of food plant species, and the quality of the plants used affects the development time and the absolute values of at least the eyespot patterns in the laboratory stock of *B. anynana*, but the shape of the reaction norm is unchanged (Kooi et al. 1996). We also know that the same is true when comparing thermal responses of *B. anynana* populations from different locations across its range (de Jong et al. 2010). When interpreting the results from our study it is therefore important to put the emphasis on the shape of the reaction norm rather than focusing on differences in absolute values (except very large differences).

### Statistical analyses

Two-way ANOVAs were used to analyze the effect of developmental temperature and sex on each phenotypic trait of interest, initially fitting full models including temperature, sex, and their interaction as fixed factors and removing nonsignificant terms successively. Minimum adequate and full models are presented in Table 1 and Supplementary Table 1, respectively. Abdomen ratio was computed by dividing abdomen dry mass by total adult dry mass, and this measure was arcsine transformed prior to statistical analysis. RMR and eyespot size were first corrected for body or wing size prior to analysis in the two-way ANOVAs. This was done by first fitting separate linear regression models on each of those traits with adult dry mass (in the case of RMR), or wing size (in the case of eyespot size) as sole predictor variable. The residuals of each of these models were analyzed as dependent variables in the two-way ANOVAs. In all ANOVAs, the residuals were normally distributed as evaluated visually using Q-Q plots.

**Table 1:** Minimum adequate models of the effect of developmental temperature and sex on a suite of phenotypic traits in *Bicyclus sanaos* and *B. anynana*, related to Figures 6. In one case where none of the fixed effects was significant (*P* < 0.05), the full model is shown (marked with an *). See Supplementary Table 1 for full models of all traits. The data for *B. anynana* have previously been used in another study Oostra et al. (2011)

Species	Dependent variable	Fixed effects	*F*	df	*P*
*B. sanaos*	Total development time	Temperature	356.69	2,202	<0.00001
*B. anynana*	Total development time	Sex	46.49	1,830	<0.00001
		Temperature	3443.87	2,830	<0.00001
		Sex × Temperature	5.88	2,830	0.00292
*B. sanaos*	Larval development time	Temperature	201.54	2,202	<0.00001
*B. anynana*	Larval development time	Sex	170.39	1,830	<0.00001
		Temperature	3999.71	2,830	<0.00001
		Temperature × Sex	15.26	2,830	<0.00001
*B. sanaos*	Pupal development time	Sex	34.69	1,202	<0.00001
		Temperature	2224.42	2,202	<0.00001
*B. anynana*	Pupal development time	Sex	16.12	1,832	0.00006
		Temperature	780.42	2,832	<0.00001
B. sanaos*	Pupal mass	Sex	0.05	1,200	0.8253
		Temperature	0.32	2,200	0.7283
		Temperature × Sex	0.09	2,200	0.9159
*B. anynana*	Pupal mass	Sex	1262.0	1,832	<0.00001
		Temperature	54.21	1,832	<0.00001
*B. sanaos*	Adult dry mass	Sex	87.21	1,203	<0.00001
		Temperature	4.32	2,203	0.01458
*B. anynana*	Adult dry mass	Sex	3801.5	1,305	<0.00001
		Temperature	16.89	1,305	<0.00001
*B. sanaos*	Size-corrected RMR	Sex	106.27	1,191	<0.00001
		Temperature	21.19	2,191	<0.00001
*B. anynana*	Size-corrected RMR	Temperature	87.58	2,186	<0.00001
		Temperature × Sex	4.73	2,186	0.00332
*B. sanaos*	Abdomen ratio (arcsine transformed)	Sex	1122.0	1,205	<0.00001
*B. anynana*	Abdomen ratio (arcsine transformed)	Sex	1299.71	1,303	<0.00001
		Temperature	20.842	2,303	<0.00001
		Temperature × Sex	26.27	2,303	<0.00001
*B. sanaos*	Size-corrected second eyespot radius	Sex	12.23	1,187	0.00059
		Temperature	34.98	2,187	<0.00001
*B. anynana*	Size-corrected second eyespot radius	Sex	18.91	1,214	0.00002
		Temperature	171.01	2,214	<0.00001
*B. sanaos*	Size-corrected fifth eyespot radius	Sex	90.70	1,187	<0.00001
		Temperature	67.05	2,187	<0.00001
*B. anynana*	Size-corrected fifth eyespot radius	Sex	4.75	1,212	0.03049
		Temperature	170.43	2,212	<0.00001
		Temperature × Sex	3.97	2,212	0.02034

Mortality was compared between the three temperature treatments by analyzing egg to adult survival using Chi-squared tests. All analyzes were performed in the R statistical environment (R Development Core Team 2010).

## Results

### Low survival at lowest temperature

Larvae and pupae performed significantly worse at the lowest temperature. Egg to adult survival rate was 20% at 19°C, 42% at 23°C, and 41.5% at 27°C (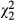 = 18.29, *N* = 207, *P* = 0.0001). Assuming an equal sex ratio among the hatching larvae, females had a slightly higher mortality across temperatures (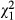 = 4.06, *N* = 207, *P* = 0.04). The total number of females eclosing was 89 and for the males 118.

### Development time and lack of protandry

**Figure 1:**
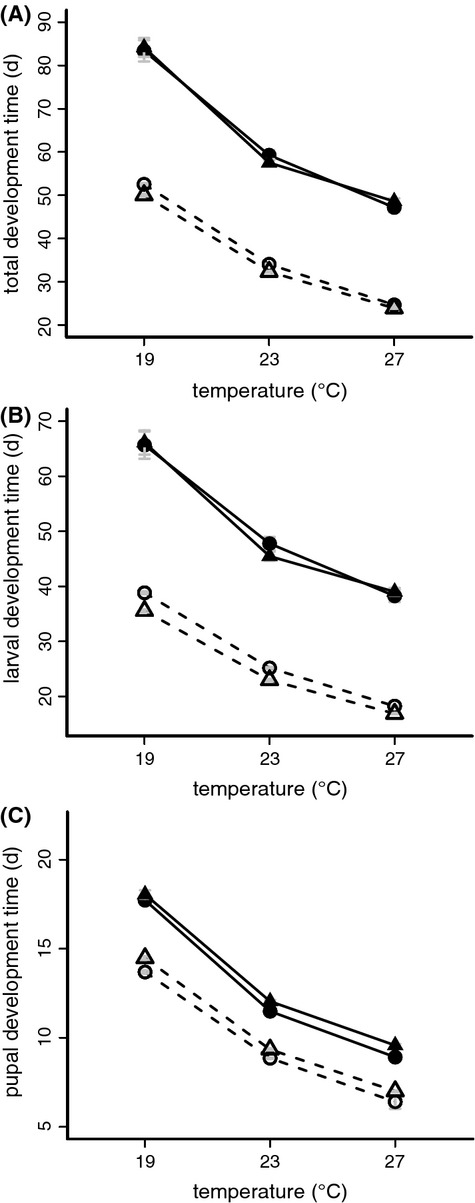
Development time for *Bicyclus sanaos* (solid lines) and *B. anynana* (dashed lines) shown as total (A) developmental time, and broken up in larval (B) and pupal (C) time. Both species are affected by temperature with slower development in cold conditions. In *B. anynana,* there is also strong protandry, with males (triangles) emerging before females (circles), a pattern not seen in *B. sanaos*. See Table 1 and Supplementary Table 1.

Total egg to adult development time in *B. sanaos* was strongly affected by developmental temperature, as indicated by the shape of reaction norms and wide differences between extreme temperatures (Table 1; Fig. 1). Both females and males developed faster at higher temperatures but there was no evidence for protandry in *B. sanaos*. The sexes developed at the same rate and showed the same temperature response. This contrasts sharply with the protandry shown by *B. anynana* across all temperatures, especially at the higher temperatures (Fig. 1A). Overall, *B. sanaos* developed much slower than *B. anynana*. Examining larval and pupal development time separately revealed that the lack of protandry in *B. sanaos* originated in the larval stage. The average duration of the larval stage was equal for females and males, while in *B. anynana* male larvae develop significantly faster than female larvae (Fig. 1B). In contrast, during the pupal stage females of both species developed faster than males (Fig. 1C). The latter effect was small and did not affect total development time.

### Plasticity and sexual dimorphism in body size

**Figure 2:**
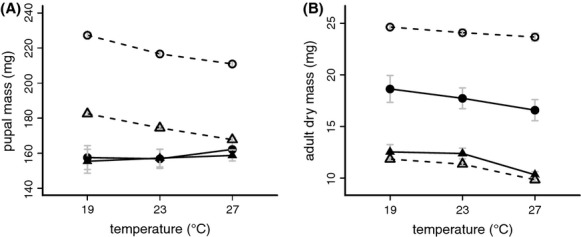
Pupal mass (A) and adult dry mass (B) in *Bicyclus sanaos* (solid lines) and *B. anynana* (dashed lines) as a function of developmental temperature for females (circles) and males (triangles). Both species produce smaller adults at warmer developmental temperatures, and females are consistently larger than males. For *B. sanaos,* the pupal mass is not affected by either rearing temperature or sex. See Table 1 and Supplementary Table 1.

Despite large changes in development time in response to temperature, pupal mass of *B. sanaos* did not differ across developmental temperatures. Furthermore, there was no sexual dimorphism in this trait (Table 1; Fig. 2A). In contrast, adult dry mass showed a significant temperature response, with a larger size at lower temperatures, and females were consistently larger than males (Fig. 2B). In *B. anynana*, both pupal and adult dry masses are affected by developmental temperature (Fig. 2). Furthermore, female *B. sanaos* pupae developed to become larger adults than male pupae of similar mass. Thus, in *B. sanaos*, in contrast to *B. anynana*, temperature plasticity of body size and sexual size dimorphism are only detectable in adults, and, therefore, originate in the pupal stage. Although the same average weight is accumulated during the larval stage, male pupae lose more weight than female pupae during metamorphosis and end up as smaller adults. Pupae developed at higher temperatures lose more mass during the pupal stage than those developed at lower temperatures and do so in a shorter time period (cf. Fig. 1C).

### Imprint of developmental temperature on adult RMR

**Figure 3:**
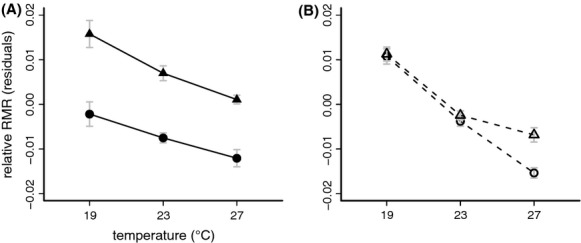
Mass-corrected adult RMR (ml CO_2_ hr^−1^; see Methods) in *Bicyclus sanaos* (A) and *B. anynana* (B) as a function of developmental temperature for females (circles) and males (triangles). The difference between the sexes is observed in *B. sanaos* is only present at 27 °C for *B. anynana*. See Table 1 and Supplementary Table 1.

Developmental temperature had a significant effect on mass-corrected adult RMR, with females and males reared at low temperatures expressing higher RMR as adult. Furthermore, across all temperatures males had a higher RMR than females (Table 1; Fig. 3A). *Bicyclus anynana* showed a similar temperature imprint with mass-corrected adult RMR being higher in individuals reared at lower temperatures. However, males only had higher RMR than females at 27°C (Fig. 3B).

### Reduced plasticity in reproductive body allocation

**Figure 4:**
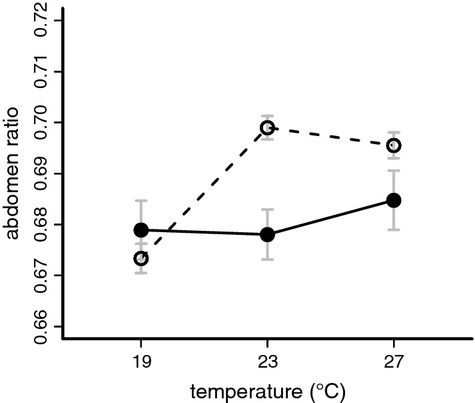
Abdomen ratio (abdomen dry mass divided by total dry mass) in *Bicyclus sanaos* (solid lines) and *B. anynana* (dashed lines) females as a function of developmental temperature. Females of *B. anynana* put more resources into abdominal tissue when developing at high temperatures, while no such effect is found for females of *B. sanaos*. See Table 1 and Supplementary Table 1.

We determined abdomen ratio (relative allocation of adult body mass to abdomen) as a measure of reproductive investment (cf. Oostra et al. 2011). There was no effect of temperature on abdomen ratio in *B. sanaos* (Table 1, Supplementary Table 1). In contrast, abdomen ratio in *B. anynana* was significantly affected by temperature, with relatively larger abdominal sizes at higher temperatures. The difference between the females of the two species is shown in Fig. 4. These data indicate a lack of developmental plasticity of allocation to abdomen in *B. sanaos*, contrasting with the high temperature sensitivity observed in *B. anynana*.

### Phenotypic plasticity of wing pattern

**Figure 5:**
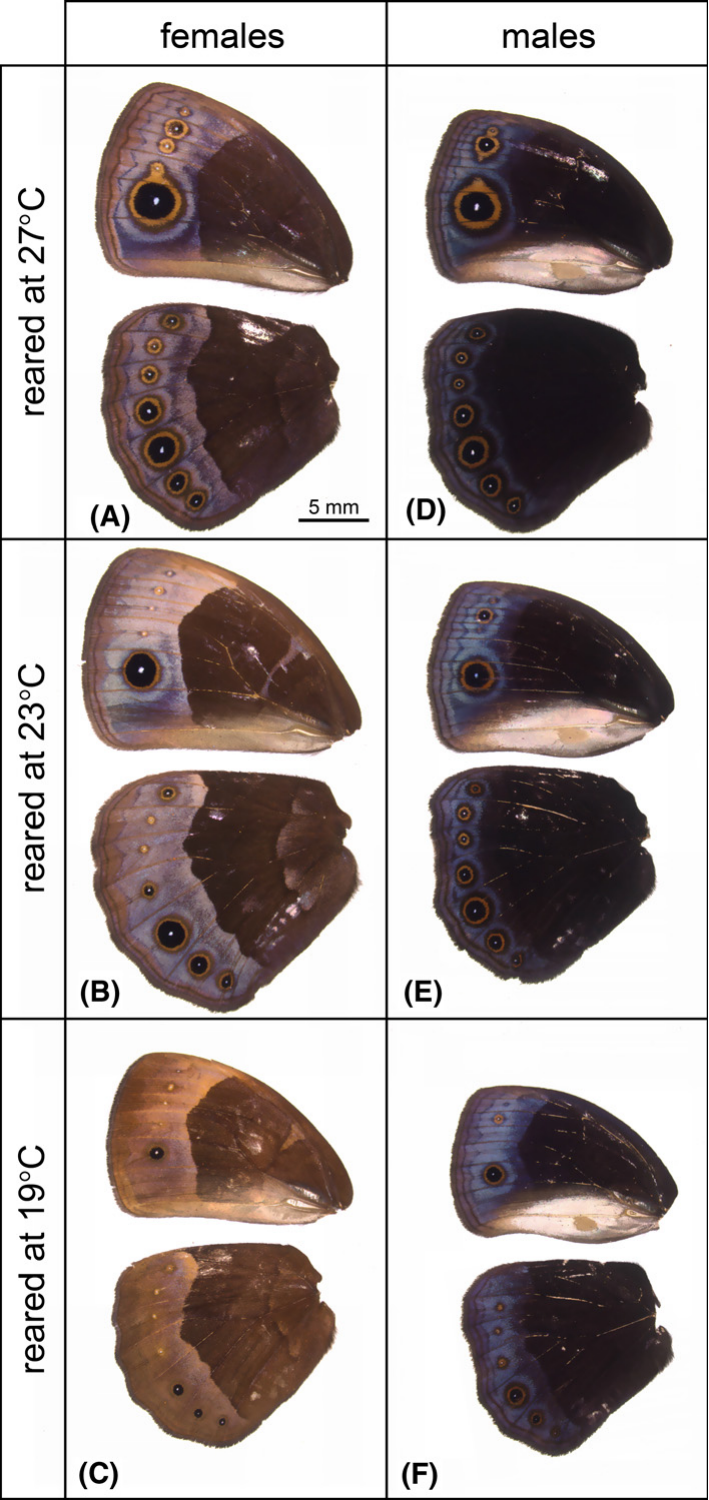
Wing patterns of adult *Bicyclus sanaos* reared at different temperatures as larvae. The ventral surfaces of fore, – and hindwings of representative *B. sanaos* females (left) and males (right) reared at 27 (A, D), 23 (B, E), or 19°C (C, F) are shown. More details of the quantitative phenotypic difference are shown in figure 6.

**Figure 6:**
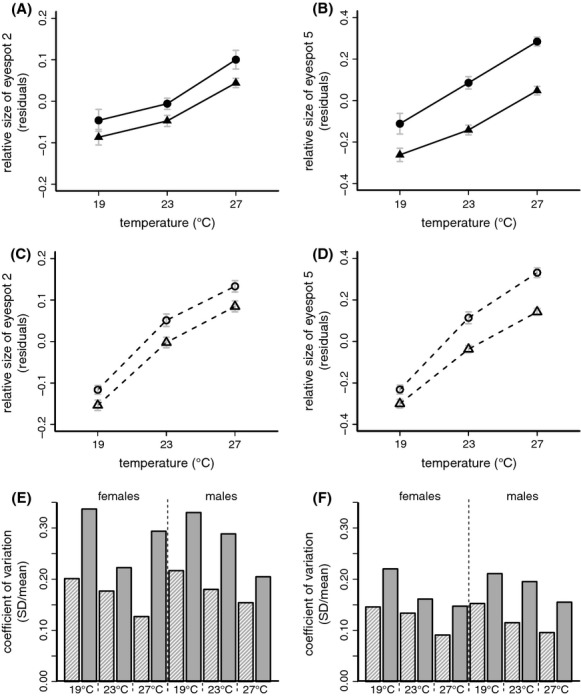
The relative size of two measured eyespots for *Bicyclus sanaos* (A, B) and *B. anynana* (C, D) on the ventral hindwing (mm; see Methods) as a function of developmental temperature, for females (circles) and males (triangles). Both species show a strong effect of temperature with smaller eyespots when reared in colder conditions. Females have consistently larger eyespots than males (See Table 1 and Supplementary Table 1). Investigating the coefficients of variation (standard deviation/mean) in the size of the second (E) and fifth (F) eyespot in *B. sanaos* (filled bars) and *B. anynana* (striped bars) in females and males at all three developmental temperatures (equal sample sizes for both species; see Methods) reveals higher variation at all temperatures for *B. sanaos*.

The ventral wing patterns of females and males of both species showed a marked response to developmental temperature. Both the sizes of the eyespots as well as coloration of the wing differed substantially between cohorts reared at different temperatures (Fig. 5). We quantified these differences for two eyespots and found that the size of the second (Fig. 6A and C) and fifth (Fig. 6B and D) eyespot on the ventral hind wing (corrected for wing size) was strongly affected by developmental temperature (Table 1). Both species had smaller eyespots when reared at lower temperatures and females generally had larger spots. The coefficients of variation (standard deviation divided by the mean per species per sex per temperature) were on average *ca*. 50% higher in *B. sanaos* compared to *B. anynana*, and at all temperatures variation was highest in *B. sanaos* in each sex (Fig. 6E and F). Taken together, we show that eyespot size is a phenotypically plastic trait in *B. sanaos*. It responds in the same direction and to the same extent to developmental temperature as in its seasonal congener *B. anynana*: a lower temperature during development induces the expression of smaller eyespots in adults. Although both species show plasticity in wing pattern, variation in eyespot size is consistently higher in *B. sanaos* compared to *B. anynana*. Data (Oostra et al 2014a) available from the Dryad Digital Repository.

## Discussion

The rainforest butterfly *B. sanaos* showed striking differences in thermal responses among traits that in *B. anynana* are all highly responsive to developmental temperature and are involved in seasonal adaptation. Most traits (development time, adult mass, RMR, and ventral eyespot size), responded readily to temperature, albeit not always in exactly the same way as in *B. anynana* (Figs. 3 and 6). The observed temperature plasticity in these traits was in stark contrast with the lack of response to temperature for relative abdomen size. In *B. anynana*, adults develop a relatively larger abdomen when reared under warm, wet season conditions, reflecting the higher early reproductive investment in this season (Oostra et al. 2011). However, in *B. sanaos* this was not the case: females showed no evidence of increased mass allocation to abdomen when reared in warm conditions, with the slope of thermal reaction norms not deviating from zero (Fig. 4). The same was true for the males. Thus, exposing *B. sanaos* in the laboratory to a range of temperatures the species not normally would be exposed to in nature reveals plasticity for some traits, but not all.

### Mechanisms of life-history integration

In many animals, plastic traits are integrated into functional suites that covary in response to environmental cues (Schlichting and Pigliucci 1998; Pigliucci 2003; Brakefield and Zwaan 2011; Simpson et al. 2011). Shared endocrine regulation plays a key role in accomplishing such phenotypic integration of multiple traits and adjusting them in a coordinated and timely fashion (Denlinger 2002; Ketterson et al. 2009). In *B. anynana*, several seasonally plastic traits investigated in this study are controlled by a single hormonal system. In particular, Ecdysteroid hormones active during the pupal stage control pupal development time (Zijlstra et al. 2004) and mediate developmental plasticity of the ventral wing pattern (Koch et al. 1996; Brakefield et al. 1998), female relative abdomen size, and female reproductive strategy (Oostra et al. 2014b). Pupal development time and wing pattern show strong genetic and phenotypic correlations, due to sharing the same underlying hormonal regulator. At the same time, they also express substantial trait-independent genetic variation, and antagonistic selection targeting this variation is able to uncouple the two traits (Zijlstra et al. 2003, 2004). A similar scenario could explain the differences in plasticity between wing pattern and abdomen size as observed in *B. sanaos*, which in *B. anynana* both show plasticity and share a single hormonal regulator. These traits may have previously been hormonally coupled in the ancestral lineage of *B. sanaos* inhabiting the seasonal savannah. Divergent selective pressures for these traits in the less seasonal rainforest habitat (see below) in combination with trait-independent genetic variation may have resulted in their decoupling over evolutionary time. Hormonal manipulations in *B. sanaos* could reveal whether abdomen size has indeed lost its plasticity due to decoupling from environmentally sensitive hormonal regulation.

### Adaptive value of body mass allocation

The allocation of body mass to the abdomen is an important determinant of female reproductive investment and early fecundity in many insects, including Lepidoptera (Boggs 1981; Jervis et al. 2005; Kivela et al. 2012). Any (plastic) reduction in female abdomen size would thus likely inflict a strong cost for species under time constraints (Brakefield et al. 2001). For *B. anynana*, the dry season in the savannah is a period of severely limited reproductive opportunities, due to lack of larval food plants (Brakefield and Zwaan 2011). Under such circumstances, selective pressures in the dry season likely drive the seasonally plastic reallocation of mass away from the abdomen, as observed in the laboratory (Saastamoinen et al. 2010). In contrast, the rainforest species *B. sanaos* does not experience a seasonal reduction in food availability, as green larval food plants continue to be abundant even at the end of the dry season. Therefore, it has the potential to breed continuously throughout the year, with overlapping generations. This would relax the need to invest larval-derived resources in a large abdomen already at eclosion in order to be able to start ovipositing very early in life (potentially at the expense of lifetime fecundity). Indeed, *B. sanaos* females in the laboratory start ovipositing late, continue to do so for a long time and live relatively long lives compared to *B. anynana* (pers. obs.). In addition, a relatively heavy abdomen could potentially constrain adult flight ability, increasing susceptibility to predation (cf. Srygley and Chai 1990). Together, this could explain why the ratio of abdomen to thorax mass at eclosion was more similar to that in *B. anynana* in the dry, not the wet season (Fig. 4). If *B. sanaos* females would still have the developmental machinery for reduced mass allocation to abdomen in response to temperature, occasional colder periods could inflict a serious cost on fecundity by further reducing an already decreased allocation to the abdomen. This loss of temperature sensitivity in abdominal allocation might therefore have evolved via selection for maintaining a stable abdomen size despite occasional temperature fluctuations.

### Wing patterns and predation risk

Seasonal plasticity of eyespot size in *Bicyclus* butterflies is maintained by opposing forces of natural selection in the dry and wet season savannah environments (Brakefield and Frankino 2009). As a rainforest species from a constant, green environment, *B. sanaos* is not exposed regularly to a dry season where reduced eyespots would be beneficial, but is instead under constant selection favoring large, deflective eyespots. In absence of other major evolutionary forces, such a situation would presumably lead to reduced phenotypic plasticity in wing pattern. However, this is contrary to our current findings. One explanation for the evolutionary maintenance of eyespot size plasticity in *B. sanaos* could be that temperature sensitivity is still beneficial within the green rainforest environment. Higher temperatures permit higher flight activity (e.g., via increased metabolic rate; Niitepold et al. 2009) but this may carry enhanced predation risks (cf. Bonte et al. 2012). If large eyespots are more effective in deflecting predatory attacks than small ones, expressing large eyespots might permit higher levels of activity at higher temperatures. However, such temperature-dependent fitness consequences of variation in eyespot size within the rainforest (or during the wet season on the savannah) are probably very subtle, making it very difficult to test this hypothesis. Empirically establishing the selective advantage of wet season-like eyespots as deflectors of predator attacks has been notoriously difficult, contrasting with the firmly established strong selection for crypsis in the dry season (Brakefield and Frankino 2009).

If indeed there is no strong selection acting on wing pattern plasticity within the rainforest environment, constraints on evolutionary change toward reduced plasticity might be more important. In this hypothesis, the evolutionary retention of eyespot size plasticity in *B. sanaos* is best explained as a legacy of past selection in a seasonal environment. In *B. anynana*, it has been shown that a lack of temperature-independent genetic variation in eyespot size can constrain the evolution of reduced plasticity, at least in the short term. Artificial selection experiments targeting the slope of the reaction norm for wing pattern failed to produce lines with reduced plasticity. This was due to high, positive genetic correlations across temperatures (Wijngaarden and Brakefield 2001; Wijngaarden et al. 2002). The observed retention of wing pattern plasticity in *B. sanaos* indicates that such strong developmental constraints may also be relevant over longer evolutionary time scales, especially if selective benefits of wing pattern plasticity in the rainforest environment are weak. Although we did not measure genetic variation, phenotypic variation for eyespot size was substantially higher in *B. sanaos* compared to *B. anynana*, and more so at lower temperatures (Fig. 6E and F), consistent with the view of relaxed selection on eyespot size and/or increased mutational variation.

### Protandry, sexual size dimorphism, and size plasticity

In seasonal insects, time constraints on development can promote the evolution of protandry, while continuous breeding opportunities with overlapping generations are associated with absence of protandry (Nylin et al. 1993; Blanckenhorn et al. 2007; Allen et al. 2011). *Bicyclus sanaos*, inhabiting an environment with limited seasonal variation in reproductive opportunities, showed no evidence of protandry: males did not develop faster than females at any temperature (Fig. 1A). This contrasts sharply with the significant protandry observed in a number of previous experiments in *B. anynana* (e.g., Oostra et al. 2011), which only has a limited time period in the wet season to reproduce. In this species, artificial selection experiments showed that strong genetic correlations in development time between the sexes preclude all but a small short-term evolutionary response to selection for increased or decreased protandry (Zwaan et al. 2008). This is likely the result of strong selection in the past on protandry, reducing sex-independent genetic variation for development time (Allen et al. 2011). The lack of protandry observed in *B. sanaos* suggests that such a genetic constraint can be broken over longer evolutionary time scales.

As in the majority of insects (Stillwell et al. 2010), *B. sanaos* adults showed female-biased sexual size dimorphism (Fig. 2B). However, this was not yet the case in the pupal stage (Fig. 2A), when males and females were equally large. This indicates that, in contrast to many insects (Allen et al. 2011), female and male *B. sanaos* larvae grow equally fast, reaching the same pupal mass at the same time (Fig. 1B). Subsequently, during the pupal stage, female pupae lose less mass and end up being larger as adult than males. The higher RMR observed in adult males compared to females (Fig. 3A) together with the longer pupal development time (Fig. 1C) suggests that pupal development consumes more resources in males compared to females.

A similar pattern was observed for thermal plasticity of pupal and adult mass: individuals reared at lower temperatures were larger as adult, but not as pupa (Fig. 2). The negative effect of developmental temperature on adult size observed in many ectotherms (Atkinson 1994) is usually explained by the effect of temperature on duration and rate of growth (Davidowitz and Nijhout 2004; Edgar 2006). However, *B. sanaos* larvae developing at low temperatures reached the same average pupal mass as those developing at high temperatures, despite the much longer larval developmental time (Fig. 1B). During the pupal stage, these pupae lost less mass than those that had developed at higher temperatures, ending up as larger adults. Thus, independent of its effects on larval growth, temperature can affect metamorphosis and utilization of larval-derived resources to the adult body.

### A larval temperature signature of RMR

Previous studies in *B. anynana* (e.g., Pijpe et al. 2007) and other insects (Berrigan 1997; Le Lann et al. 2011) reported a negative effect of development temperature on adult RMR, suggesting a general mechanism for coping with lower temperatures rather than a specific seasonal adaptation. We observed the same general pattern in *B. sanaos*, but there are interesting differences between the sexes. In *B. anynana*, RMR is higher in males compared to females, but only at 27°C. This might relate to the higher reproductive activity in wet season conditions, when males are actively searching for females and fighting for territories. *B. sanaos* males have a higher RMR compared to females regardless of developmental temperature, suggesting that *B. sanaos* males do not alter breeding activity in response to temperature, similar to the lack of plasticity in female abdominal allocation (see above).

## Conclusions

Exposing the aseasonal rainforest butterfly *B. sanaos* in the laboratory to a range of temperatures not normally encountered in the field revealed hidden reaction norms for several traits, including wing pattern and adult size. In contrast, allocation of adult mass to the abdomen (a proxy for early-life reproductive investment) was not affected by developmental temperatures. In the savannah butterfly *B*. *anynana,* these traits show developmental plasticity as an adaptation to the contrasting environments of its seasonal habitat. In that species, wing pattern and allocation to abdomen respond to developmental temperature via a common hormonal system active during pupal development. Our results for *B. sanaos* strongly suggest that such shared hormonal regulation does not preclude decoupling of temperature responses between traits over evolutionary time. The observed differences in plasticity with *B. anynana* are likely the result of long-term relaxed selection on plasticity over millions of years for *B. sanaos*. Other species from similar habitats as our two study species are likely to show similar responses. However, given that we have only been able to compare two species it could be possible that differences not directly related to species-specific selective regimes might influence our results. We can identify three such alternative explanations, but find them unlikely to cause the observed differences. First, the lack of plasticity in body allocation in *B. sanaos* (with a relatively small abdomen across treatments) might be attributable to wheat being a less efficient food source, so that the larvae struggle to reach anything but a small abdominal size at eclosion regardless of rearing temperature. Having reared around ten *Bicyclus* species from across Africa in the laboratory over the last years we have observed that maize in general is a much less suitable food plant for these species than wheat. While all reared species developed well on wheat, many showed high mortality when reared on maize (O. Brattström, unpubl. data). Second, the laboratory population of *B. anynana* has been bred in laboratory conditions for more than a hundred generations (Brakefield et al. 2009). This more stable environment may be interpreted as artificially relaxed selection on plasticity, but if that were the case its effect should be in the direction of more similarities between the species compared to their presumed natural state, which is contrary to our observed results. Third, the amount of available genetic variation within our two test populations could be quite different, given differences in founding population size and time since establishment in the laboratory. However, this cannot explain why *B. sanaos* should lose plasticity in one trait only (abdomen allocation) while retaining it in the others. Considering these factors we conclude that the results of our study reflect the natural thermal response of the wild populations of the two investigated species. We hypothesize that the loss of plasticity in abdomen allocation is the result of strong natural selection against temperature-induced fecundity reduction in the rainforest, combined with selection for more continuous breeding over a longer life span. For wing pattern, such selective forces are likely much weaker, resulting in retention of developmental plasticity. This implicates that costs of plasticity in *Bicyclus* butterflies mainly stem from mismatch costs, that is, expressing a suboptimal phenotype in a particular environment (Auld et al. 2010), which likely differ between phenotypically plastic traits. Thus, hormonal integration between plastic traits – as a result of past selection on expressing a coordinated environmental response – can be broken when the optimal reaction norms for those traits diverge in a new environment. Our understanding of the extent to which plasticity in wing pattern and in life history can evolve independently would greatly benefit from studying these traits systematically in a phylogenetic context. In general, we argue that comparing phylogenetically closely related species allows for both discerning patterns of adaptive evolution in the individual species and traits as well as to follow patterns of trait loss and their adaptive significance (Ellers et al. 2012).

## Supporting Information

**Table S1.** Full models of the effect of developmental temperature, sex and their interaction on a suite of phenotypic traits in *B. sanaos* and *B. anynana*, related to Figures 1–4 & 6. See Table 1 in the main text for the minimum adequate models. The data for *B. anynana* have previously been used in another study (Oostra et al. 2011).
