# Supplementary material for: On the fate of seasonally plastic traits in a rainforest butterfly under relaxed selection
Source: Ecol Evol. 2014 Jun 4;4(13):2654–67. doi: 10.1002/ece3.1114 (PMC4113290; doi:10.1002/ece3.1114)
Supplement: Supplementary file 1 — Table S1. Full models of the effect of developmental temperature, sex and their interaction on a suite of phenotypic traits in B. sanaos and B. anynana, related to Figures 1–4 & 6. See Table 1 in the main text for the minimum adequate models. The data for B. anynana have previously been used in another study (Oostra et al. 2011). [file ece30004-2654-SD1.doc]

| **species** | **dependent variable** | **fixed effects** | **F** | **df** | **P** |
| --- | --- | --- | --- | --- | --- |
| *B. sanaos* | total development time | sex  temperature  temperature x sex | 0.13  160.96  1.14 | 1, 199  2, 199  2, 199 | 0.71900  <0.00001  0.32260 |
| *B. anynana* | total development time | sex  temperature  sex x temperature | 46.49  3443.87  5.88 | 1, 830  2, 830  2, 830 | <0.00001  <0.00001  0.00292 |
| *B. sanaos* | larval development time | sex  temperature  temperature x sex | 0.04  90.24  1.10 | 1, 199  2, 199  2, 199 | 0.83240  <0.00001  0.33520 |
| *B. anynana* | larval development time | sex  temperature  temperature x sex | 170.39  3999.71  15.26 | 1, 830  2, 830  2, 830 | <0.00001  <0.00001  <0.00001 |
| *B. sanaos* | pupal development time | sex  temperature  temperature x sex | 2.31  1033.14  0.82 | 1, 199  2, 199  2, 199 | 0.13020  <0.00001  0.44100 |
| *B. anynana* | pupal development time | sex  temperature  temperature x sex | 8.05  418.37  0.31 | 1, 830  2, 830  2, 830 | 0.00467  <0.00001  0.73218 |
| *B. sanaos* | pupal mass | sex  temperature  temperature x sex | 0.05  0.32  0.09 | 1, 200  2, 200  2, 200 | 0.82530  0.72830  0.91590 |
| *B. anynana* | pupal mass | sex  temperature  temperature x sex | 415.95  33.18  0.38 | 1, 830  2, 830  2, 830 | <0.00001  <0.00001  0.68270 |
| *B. sanaos* | adult dry mass | sex  temperature  temperature x sex | 18.35  1.34  0.23 | 1, 201  2, 201  2, 201 | 0.00003  0.26480  0.79540 |
| *B. anynana* | adult dry mass | sex  temperature  temperature x sex | 1218.27  3.55  2.82 | 1, 303  2, 303  2, 303 | <0.00001  0.03001  0.06115 |
| *B. sanaos* | size-corrected RMR | sex  temperature  temperature x sex | 30.35  5.59  0.74 | 1, 189  2, 189  2, 189 | <0.00001  0.00439  0.48013 |
| *B. anynana* | size-corrected RMR | sex  temperature  temperature x sex | 0.08  87.58  4.08 | 1, 186  2, 186  2, 186 | 0.77738  <0.00001  0.01847 |
| *B. sanaos* | abdomen ratio (arcsine transformed) | sex  temperature  temperature x sex | 221.20  0.62  1.60 | 1, 201  2, 201  2, 201 | <0.00001  0.53770  0.20450 |
| *B. anynana* | abdomen ratio (arcsine transformed) | sex  temperature  temperature x sex | 1299.71  20.84  26.27 | 1, 303  2, 303  2, 303 | <0.00001  <0.00001  <0.00001 |
| *B. sanaos* | size-corrected second eyespot radius | sex  temperature  temperature x sex | 1.78  16.84  0.14 | 1, 185  2, 1852 | 0.18400  <0.00001  0.86620 |
| *B. anynana* | size-corrected second eyespot radius | sex  temperature  temperature x sex | 3.78  91.39  0.19 | 1, 212  2, 212  2, 212 | 0.05331  <0.00001  0.82391 |
| *B. sanaos* | size-corrected fifth eyespot radius | sex  temperature  temperature x sex | 8.51  35.68  1.03 | 1, 185  2, 185  2, 185 | 0.00396  <0.00001  0.35766 |
| *B. anynana* | size-corrected fifth eyespot radius | sex  temperature  temperature x sex | 4.74  170.43  3.97 | 1, 212  2, 212  2, 212 | 0.03049  <0.00001  0.02034 |

**Supplementary table 1.** Full models of the effect of developmental temperature, sex and their interaction on a suite of phenotypic traits in *B. sanaos* and *B. anynana*, related to Figures 1-4 & 6. See Table 1 in the main text for the minimum adequate models. The data for *B. anynana* have previously been used in another study (Oostra *et al*., 2011).
